# EEG-based detection of emotional valence towards a reproducible measurement of emotions

**DOI:** 10.1038/s41598-021-00812-7

**Published:** 2021-11-03

**Authors:** Andrea Apicella, Pasquale Arpaia, Giovanna Mastrati, Nicola Moccaldi

**Affiliations:** 1 Laboratory of Augmented Reality for Health Monitoring (ARHeMLab), Department of Electrical Engineering and Information Technology, University of Naples Federico II, Naples, Italy; 2 Interdepartmental Center for Research on Management and Innovation in Healthcare (CIRMIS), University of Naples Federico II, Naples, Italy

**Keywords:** Biomedical engineering, Emotion

## Abstract

A methodological contribution to a reproducible *Measurement of Emotions* for an EEG-based system is proposed. *Emotional Valence* detection is the suggested use case. Valence detection occurs along the *interval scale* theorized by the *Circumplex Model* of emotions. The binary choice, positive valence vs negative valence, represents a first step towards the adoption of a metric scale with a finer resolution. EEG signals were acquired through a 8-channel dry electrode cap. An implicit-more controlled EEG paradigm was employed to elicit emotional valence through the passive view of standardized visual stimuli (i.e., Oasis dataset) in 25 volunteers without depressive disorders. Results from the *Self Assessment Manikin* questionnaire confirmed the compatibility of the experimental sample with that of *Oasis*. Two different strategies for feature extraction were compared: (i) based on a-priory knowledge (i.e., Hemispheric Asymmetry Theories), and (ii) automated (i.e., a pipeline of a custom 12-band Filter Bank and Common Spatial Pattern). An average within-subject accuracy of 96.1 %, was obtained by a shallow Artificial Neural Network, while *k*-Nearest Neighbors allowed to obtain a cross-subject accuracy equal to 80.2%.

## Introduction

The word emotion derives from the Latin “Emotus” which means to bring out. Technically, emotion is the response to imaginary or real stimuli characterised by changes in individual’s thinking, physiological responses, and behaviour^1^. In the *Circumplex Model*^2^ of emotion, *Valence* denotes how much an emotion is positive or negative. A further approach to the study of emotions is provided by the *Discrete Model* of emotions (anger, fear, joy,...).

Discrimination of emotional valence is a broad issue widely addressed in recent decades, affecting the most varied sectors and finding application in multiple domains. Some application fields are for example, car driving^3,4^, working^5^, medicine^6,7^, and entertainment^8^.

Several biosignals have been studied over the years for emotions recognition: cerebral blood flow^9^, electroculographic (EOG) signals^10^, electrocardiogram, blood volume pulse, galvanic skin response, respiration, phalanx temperature^11^. In recent years, several studies have focused on the brain signal. There are many invasive and non-invasive techniques for understanding the brain signals such as: PET (Positron Emission Tomography), MEG (Magneto Encephalography), NIRS (Near-infrared Spectroscopy), fMRI (Functional Magnetic Resonance Imaging), EROS (Event-related optical signal), EEG (Electroencephalogram). Among the mentioned systems, EEG offers a better temporal resolution. There are already some portable EEG solutions on the market. Currently, a scientific challenge is to use dry electrodes^12,13^ and increasingly reduce the number of channels to maximise the user comfort while maintaining high performances.

The measurement of emotions^14^ is different from the emotion recognition and it requires the use of an *interval scale* besides the management of the reproducibility problem. The well-assessed taxonomy given by Stevens^15^ provided a fourfold classification scheme of measurement scales: *nominal*, *ordinal*, *interval*, and *ratio* scales. Nominal and ordinal scales represent non-additive quantities and, therefore, cannot be considered for measurements according to the International Vocabulary of Metrology^16^. Studies adopting the theory of discrete emotions^17^ employ a nominal scale providing only classifications. Conversely, the Circumplex Model allows the measurement of emotions by arranging them along interval scales. As concerns the second condition, often, the same stimulus or environmental condition does not induce the same emotion in different subjects (cross-subject reproducibility loss). Furthermore, the same individual exposed to the same stimulus but after a certain period of time, reacts in a different way (within-subject reproducibility loss). In psychology research, suitable sets of stimuli were validated experimentally by using significant samples and are widely used by clinicians and researchers^18^. In particular, several stimuli datasets were produced referring to the Circumplex Model and their scores were arranged along an interval scale. However, the problem of standardizing the induced response remains still open, also considering, for example, the issue of the cross-cultural generality of perceptions. The effectiveness of the emotion induction can be verified by means of self-assessment questionnaires or scales. The use of the validated stimulus rating and the subject’s self-assessment can represent an effective strategy towards the construction of a metrological reference for the EEG-based reproducible measurement of emotions^19^. Furthermore, the use of assessment tools during the sample construction can soften possible emotional bias caused by psychiatric disorders. As concerns the measurement model, older approaches predominantly made use of a priori knowledge. Emotions studies, based on spatial distribution analysis of EEG signal, were principally focused on the asymmetric behaviour of the two cerebral hemispheres^20–22^. Two theories, in particular, model the relationship between emotions and asymmetry in a different way. The *Theory of Right Hemisphere* posits that the right hemisphere is dominant over the left hemisphere for all forms of emotional expression and perception. Instead, the *Theory of Valence* states that the right hemisphere is dominant (in term of signal amplitude) for negative emotions and the left hemisphere is dominant for positive emotions. In particular the theory of valence focuses on what happens in the two areas of the prefrontal cortex. The prefrontal cortex plays an important role in the control of cognitive functions and in the regulation of the affective system^23^. The EEG asymmetry allows to evaluate the subject’s emotional changes and responses and, therefore, it can serve as an individual feature to predict emotional states^24^.

The most common frequency index for emotion recognition is the so called *frontal alpha asymmetry* ($\alpha _{asim}$)^25^:

1 $$\begin{aligned} \alpha _{asim} = \ln (\alpha _{PSD_L})-\ln (\alpha _{PSD_R}) \end{aligned}$$

where the parameters $\alpha _{PSD_L}$ and $\alpha _{PSD_R}$ are the power spectral densities of the left and right hemispheres in the alpha band. Frontal alpha asymmetry could also predict emotion regulation difficulties by resting state EEG recordings. Frontal EEG asymmetry effects are quite robust to individual differences^26^.

Several modern Machine Learning systems automatically carry out the feature extraction procedure. Therefore, a very large number of data from different domains (i.e., spatial, spectral or temporal) can be used as input to the classifier without an explicit hand-crafted feature extraction procedure.

Spatial filters usually enhance sensitivity to particular brain sources, to improve source localization, and/or to suppress muscular or ocular artifacts^27^. Two different categories of spatial filters exist: those dependent on data and those not dependent on data. Spatial filters not dependent on data (i.e., Common Average Reference, Surface Laplacian spatial filters) generally use fixed geometric relationships to determine the weights of the transformation matrix. The data-dependent filters, although more complex, allow better results for specific applications because they are derived directly from user’s data. They are particularly useful when little is known about specific brain activity or when there are conflicting theories (i.e., theory of valence and theory of the right hemisphere).

The aim of this research is to improve the reproducibility of a valence EEG-based emotion detection method. The reference theory adopted allows the measurement of emotions arranging them along interval scales. The architecture, designed for everyday applications, exploits a low number of data acquisition channels (i.e., 8) and dry electrodes. In “Related works” section, a State of Art of emotional valence detection is reported. The statement of the metrological problem for the EEG-based emotion assessment is presented in “ Statement of the metrological problem” section. In “Proposal” section, the basic ideas, the architecture and the data analysis of the proposed method are highlighted. Then, in “Experiments and results” section, the laboratory test procedure, a statistical comparison between stimuli scores and participants perceptions, and the experimental validation are reported, by detailing and by discussing the results of the compared methods.

## Related works

In this section, a State of the Art of the principal works related to emotion detection is reported. All the collected works exhibited at least an experimental sample of 10 subjects. Samples with number of subjects below this threshold were considered not statistically significant. The reported studies are organised in two subsections according to the used dataset: public (Studies based on public datasets” section) and self-produced (“Studies based on self-produced datasets” section). A further “Influencing factors of the experimental conditions” section collects analysis on the influencing factors of the experimental setup for the emotion assessment.

### Studies based on public datasets

Studies claiming the best accuracy on emotional valence assessment are based on public EEG signal datasets: SEED^28–33^, DEAP^29–32,34–44^, and DREAMER^33,41,42^.

SJTU Emotion EEG Dataset (SEED)^45,46^ is a collection of EEG signals provided by the Center for Brain-like Computing and Machine Intelligence (BCMI laboratory) of the Shanghai Jiao Tong University. EEG data were acquired while 15 participants watched 15 film clips, of about 4 min, eliciting positive, neutral, and negative emotions. The videos were selected in order to be understood without explanation, thus an implicit emotion recognition task was employed. The experiment, made of 15 trials, was repeated in 3 different days and EEG signals were recorded through the 62-channel Neuroscan system. Participants filled in the self assessment questionnaire immediately after each trial to report their emotional reactions.

The Dataset for Emotion Analysis using EEG, physiological and video signals (DEAP)^47,48^ is a multimodal dataset developed to analyse human affective states. The EEG and peripheral physiological signals of 32 participants, watching 40 one-minute long music videos were recorded. The EEG signals were acquired through the 32-channel BioSemi device. Participants were informed about the purpose of the experiment, but not further instructions were given, indeed, the emotion recognition task was implicit. Each video was rated in terms of arousal, valence, like/dislike, dominance and familiarity.

The Database for Emotion Recognition through electroencephalogram (EEG) and electrocardiogram (ECG) Signals from Wireless Low-cost Off-the-Shelf Devices (DREAMER)^49,50^ is a multimodal database recorded during emotional elicitation by means of audio-visual stimuli. 18 film clips were employed to elicit: amusement, excitement, happiness, calmness, anger, disgust, fear, sadness, and surprise. The film clips are long between 65 and 393 s. 23 participants undertook the experiment. Details about the experimental procedure were provided to participants and the rating scales used for emotional assessment were explained. An implicit emotion recognition task was performed since the subjects were not required to get into the target emotional state. Volunteers rated their affective states in terms of valence, arousal, and dominance. EEG signals were captured using the 14-channel Emotiv Epoc$+$.

A multichannel EEG emotion recognition method based on a Dynamical Graph Convolutional Neural Network (DGCNN) was proposed by Song et al^33^. Experiments were conducted on the 62-channels dataset SEED^51^ and on the 14-channels dataset DREAMER^49^. The average accuracies of 90.4 % and 79.95 % were achieved on the SEED dataset for within-subject and cross-subject settings respectively, in a three classes emotion recognition. The average accuracy of 86.23 % was obtained on valence dimension (positive or negative) of the DREAMER dataset in the within-subject configuration.

A Multi-Level Features guided Capsule Network (MLF-CapsNet) was employed by Liu et al. for a multi-channel EEG-based emotion recognition^41^. Valence (positive or negative) was classified with an average accuracy of 97.97 % on the 32-channels DEAP^47^ dataset and 94.59 % on the 14-channels DREAMER dataset. Within-subject experiments were performed. Comparable results were obtained by applying an end-to-end Regional-Asymmetric Convolutional Neural Network (RACNN) on the same datasets in a within-subject setup^42^.

### Studies based on self-produced datasets

EEG signal, acquired through ad hoc experimental activities, are employed in further studies^35,52,53^. The main stimuli used to elicit emotions in human subjects are: (i) projection of standardized sets of emotionally arousing images; (ii) viewing audio visuals; (iii) listening to music or sounds; and (iv) recall of autobiographical events. Below, the focus is mainly on studies using standardized image sets (i.e. International Affective Picture System (IAPS)^18^, and Geneva Affective Picture Database (GAPED)^54^). The use of a set of normative emotional stimuli (each image is rated according to the valence, arousal and dominance levels) enables to select stimuli eliciting a specific range of emotions.

Mehmood et al. used stimuli from the IAPS dataset to elicit positive or negative valence in 30 subjects^53^. The EEG signals were recorded via an 18 electrolyte gel filled electrodes caps. A feature extraction method, using Hjorth parameters, was implemented. A 70 % cross-subject accuracy was reached using a SVM classifier. Self-assessment tests were not administered to subjects.

More recently, several studies focused on channel reduction for improving the wearability of the emotion detection systems^55–64^.

Marín-Morales at al. designed virtual environments to elicit positive or negative valence^63^ . Images from IAPS dataset were used as stimuli. The emotional impact of the stimulus was evaluated using a SAM questionnaire. A set of features, extracted from EEG and ECG signals, was input into a Support Vector Machine classifier obtaining a model’s accuracy of 71.21 % along the valence dimension (binary classification problem). A 10-channel device was used to record the EEG signal from 15 subjects. Sensors’ foams were filled with Synapse Conductive Electrode Cream.

The EEG signals of 11 subjects were used to classify valence (positive and negative) by the authors^57^. Pictures from GAPED dataset were used as elicitative stimuli. The accuracy rates of a SVM classifier were 85.41 % and 84.18 % using the whole set of 14 channels and a subset of 10 channels respectively, in the cross-subject setting. EEG signals were acquired through a wet-14 channels device and no self-evaluation questionnaires were used.

Wei et al. proposed a real-time valence emotion detection system based on EEG measurement realized by means of a headband coupled with printed dry electrodes^64^. 12 participants undertook the experiment. Pictures selected from GAPED were used to elicit positive or negative valence. Self-evaluation questionnaires were employed. Two different combinations of 4 channels were tested. In both cases, the cross-subject accuracy was 64.73 %. The highest within-subject accuracy increased to 91.75 % from 86.83 % switching from one configuration to another. The latter two works^57,64^ both proposed the use of standardized stimuli. However, in the first one^57^, the concomitant use of self-assessment questionnaires was missing. Moreover, in the second one^64^, self-assessment questionnaires were employed but the results were not compared with the scores of the used stimuli. Failure to compare individual reactions with the standardized stimulus scores, negatively impacted on the result of the experiment.

Happy or sad emotions were elicited through images provided by the IAPS, by Ang et al^61^. The EEG signals were acquired through FP1 and FP2 dry electrodes. An Artificial Neural Network (ANN) classifier was fed with discrete wavelet transform coefficients. The best detection accuracy was 81.8 % on 22 subjects. Beyond the use of standardized stimuli, the subjects were also administered self-assessment scales. Moreover it is unclear how the SAM scores were used and whether the approach is within-subject or cross-subject.

Following two studies claiming a single-channel EEG based emotion recognition achieved employing audio-visual stimuli. Ogino et al. developed a model to estimate valence by using a single-channel EEG device^56^. Fast Fourier Transform, Robust Scaling and Support Vector Regression were implemented. EEG signals from 30 subjects were acquired and an average classification accuracy of 72.40 % was reached in the within-subject configuration. Movie clips were used to elicit emotional states and SAMs were administered to the participants for rating the valence score of the stimuli.

A cross-subject emotion recognition system based on Multilayer Perceptron Neural Network was proposed by Pandey et al^60^. An accuracy of 58.5 % was achieved in the recognition of positive or negative valence on DEAP dataset using the F4 channel.

A reduced number of channels implies a low spatial resolution. Traditional strategies for EEG signal feature extraction, combined with a-priori knowledge on spatial and frequency phenomena related to emotions, can be unusable in case of few electrodes. In a previous work of the Authors, for a single-channel stress detection instrument, a-priori spatial knowledge drove electrodes positioning^65^. However, signal processing was based on innovative and not well-settled strategies. Although proper psychometric tools were adopted for the construction of the experimental sample, the reproducibility of the experiment was adversely affected by the use of not standardized stimuli.

Further not standardized stimuli are personal memories. For example, the study^62^ presents a very interesting data fusion approach for emotion classification based on EEG, ECG, and photoplethysmogram (PPG). The EEG signals were acquired through an 8-channel device. A Convolutional Neural Network (CNN) was used to classify three emotions reaching an average accuracy for the cross-subject case of 76.94 %. However, personal memories of the volunteers were used as stimulus, compromising the reproducibility of the experimental results. Moreover, due to the adoption of the *discrete emotion* model, the study cannot be taken into account for emotion measurement goal.

### Influencing factors of the experimental conditions

In the field of emotion recognition, the use of audio-visual stimuli guarantees higher valence intensity (positive or negative) with respect to visual stimuli (pictures)^66^. Therefore, the sensitivity of the measurement system increases and the accuracy in emotion detection can be higher. However, currently there are no standardized audiovisual datasets to employ for eliciting emotions. The only exception is the dataset used by DREAMER, which contains a low number of stimuli (only 18), so penalising their randomic administration and increasing the risk of bias. Not even the most widely used EEG datasets SEED and DEAP employ a standardized stimulus dataset to elicit emotions.

Also the use of explicit rather than implicit tasks affects the effectiveness of the mood induction. Explicit instruction helps participants to get into the target emotional state, but it can be a further source of uncertainty. However, the existing standardized stimuli (IAPS, GAPED, OASIS, etc) are predominantly images characterized in an implicit setup. In order to draw on this resource and make the experiment reproducible, an implicit task, with static images, should therefore be adopted. Among the reported studies, task information is generally omitted.

Another factor that can influence the effectiveness of the emotional state induction is the way of stimuli selection. Referring to the main standardized stimuli datasets, images can be selected by choosing those with higher or lower valence scores. Polarized stimuli could increase the intensity of a certain emotional state with respect to random chosen stimuli.

For all the presented studies (i) type of stimuli, (ii) type of task, (iii) number of channels, (iv) number of participants, (v) classifier, (vi) number of classes, (vii) within-subject accuracy, and (viii) cross-subject accuracy are reported in Table 1.

The accuracy values are reported in both the within-subject and cross-subject cases, when available. In the first case, classification was carried out using data of a single subject both for training and test phases, while in the second one, classification was carried out employing the data set as a whole.

**Table 1:** Studies on emotion recognition classified according to the employed datasets (i.e. SEED, DEAP, and DREAMER), stimuli (*v*="video", *p*="picture", *m*="memories"), task (*i*="implicit", *e*="explicit", n.a.="not available"), #channels, #participants, #classes, classifiers, and accuracies (n.a.="not available").

Dataset	Study	Stimuli	Task	#channels	#participants	Classifier	#classes	Within-subject accuracy (%)	Cross-subject accuracy (%)
SEED	^28^	*v*	*i*	62	15	SincNet-R	3	94.5	90.0
	^33^	*v*	*i*	62	15	DGCNN	3	90.4	80.0
SEED & DEAP	^29^	*v*	*i*	62	15	DNN	3	n.a.	96.8
		*v*	*i*	32	32		2	n.a.	89.5
	^30^	*v*	*i*	62	15	SNN	3	n.a.	96.7
		*v*	*i*	32	32		2	n.a.	78.0
	^40^	*v*	*i*	62	15	SBSSVM	2	n.a.	72.0
		*v*	*i*	32	32		2	n.a.	89.0
	^31^	*v*	*i*	62	15	CNN	3	90.6	n.a.
		*v*	*i*	32	32		2	82.8	n.a.
	^32^	*v*	*i*	62	15	CNN	2	n.a.	86.6
		*v*	*i*	32	32		2	n.a.	72.8
DEAP	^34^	*v*	*i*	32	32	H-ATT-BGRU	2	n.a.	69.3
	^36^	*v*	*i*	32	32	CNN	2	n.a.	77.4
	^37^	*v*	*i*	4	32	LDA	2	n.a.	82.0
	^39^	*v*	*i*	32	32	LSTM-RNN	2	n.a.	81.1
	^43^	*v*	*i*	32	32	Kohonen-NN	2	76.3	n.a.
	^44^	*v*	*i*	32	32	SVM $+$ FCM	2	78.4	n.a.
	^60^	*v*	*i*	1	32	MLP	2	n.a.	58.5
DEAP & DREAMER	^38^	*v*	*i*	32	32	BioCNN	2	83.1	n.a.
		*v*	*i*	14	23		2	56.0	n.a.
	^41^	*v*	*i*	32	32	MLF-CapsNet	2	98.0	n.a.
		*v*	*i*	14	23		2	94.6	n.a.
	^42^	*v*	*i*	32	32	RACNN	2	96,7	n.a.
		*v*	*i*	14	23		2	97,1	n.a.
SELF-PRODUCED	^33^	*v*	*i*	14	23	DGCNN	2	86.2	n.a.
	^35^	*v*	*i*	19	40	MLP, KNN, and SVM	2	n.a.	90.7
	^55^	*v*	*n.a.*	1	20	MC-LS-SVM	2	n.a.	90.6
	^52^	*v*	*n.a.*	14	10	RVM	2	91.2	n.a.
	^56^	*v*	*n.a.*	1	30	SVM	2	72.4	n.a.
	^58^	*v*	*i*	1	19	*k*-NN	3	94.1	n.a.
	^59^	*p*	*e*	3	16	SVM	6	n.a.	83.3
	^57^	*p*	*n.a.*	10	11	SVM	2	n.a.	84.2
	^53^	*p*	*n.a.*	18	30	SVM	2	n.a.	70.0
	^61^	*p*	*n.a.*	2	22	ANN	2	n.a.	81.8
	^63^	*p*	*n.a.*	10	38	SVM	2	n.a.	71.2
	^64^	*p*	*n.a.*	4	12	LDA	2	86.8	64.7
	^62^	*m*	*e*	8	20	CNN	3	n.a.	76.9

## Statement of the metrological problem

The path towards the measurability of emotions still remains to be completed. In this study, some important steps are carried out to achieve this goal:

- a theoretical model compatible with emotion measurability was adopted;
- people with high scores on the Patient Health Questionnaire (PHQ) were excluded from the experimental sample in order to soften the bias of depressive disorders;
- standardized stimuli were used jointly with self-assessment questionnaires to reduce the intrinsic uncertainty of the measurand;

Nevertheless, there are still several aspects to continue working on:

- a more complete definition of an emotion model, which incorporates, for example, appropriately adjusted analyses for confounders including the impact of individual personality on the specific emotional response;
- identification of a measurement unit (enhancing the important role played in this direction by biosignals, including the EEG);
- an uncertainty analysis for identifying and weighing the sources in the measurement processes. Just to remember a few: (i) the theoretical model, (ii) the stimulus, (iii) the task, (iv) the specific individual emotional response, (v) the peculiar relationship between the individual emotional response and its manifestation in terms of neurosignal, (vi) the signal acquisition instrument, and (vii) the algorithms for signal classification.

## Proposal

This study proposes an emotional valence detection method starting from the EEG signal acquired through few dry electrodes. In this section, the basic ideas, the architecture, and the data processing of the proposed approach are presented.

### Basic ideas

Below the basic ideas are reported.

- *An EEG-based method for emotional valence detection*: Emotional functions are mediated by specific brain circuits and electrical waveforms. Therefore, the EEG signal varies according to the emotional state of the subject. However, using suitable algorithms, such a state can be recognized.
- *Low number of channels, dry electrodes, wireless connection for a good ergonomics*: An 8 channel-dry electrode device does not require a burdensome installation. The absence of the electrolytic gel eliminates the problem of residues in the hair. The good ergonomics of the instrument is also guaranteed by the absence of connection cables and, therefore, by the wireless transmission of the acquired signals. Both of them simplify the operator’s job.
- *Multifactorial metrological reference*: A multifactorial metrological reference was implemented. Images belonging to a statistically validated dataset were used as stimuli for eliciting emotions. Therefore, each image is scored according to the corresponding valence value. The metrological reference of the emotional valence is obtained by combining the scores of the stimuli (statistically founded) with the score of the self-assessment questionnaires (subjective response to the standardized stimulus). The Bland-Altman and the Spearman analysis were carried out for comparing Self-assessment questionnaires (SAM) scores and the OASIS dataset scores.
- *12–band Filter Bank*: Traditional filtering, employed to extract the information content from the EEG signals, is improved by a 12-band Filter-Bank. Compared to the five typical bands for EEG analysis (alpha, beta, delta, gamma, theta), narrowing the frequency intervals, the features resolution increases.
- *Beyond a priori knowledge*: A supervised spatial filter (namely CSP) guarantees automated feature extraction from spatial and time domains.

### Architecture

**Figure 1:**
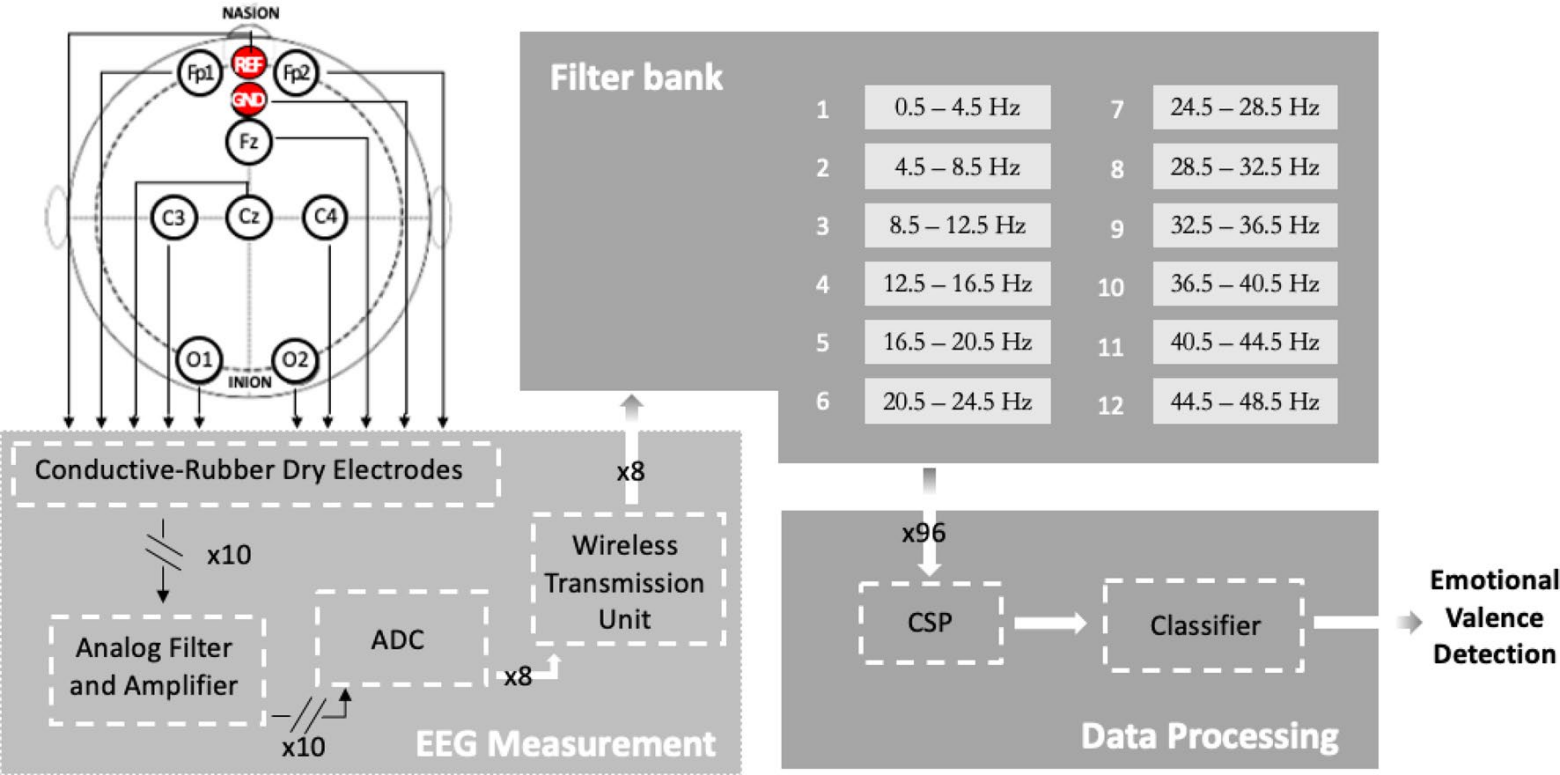
The proposed valence-detection method (CSP: Common Spatial Pattern algorithm).

The architecture of the proposed system is shown in Fig. 1. The *conductive-rubber dry electrodes* allow the EEG signals to be sensed directly from the scalp of the subject. Each channel is differential with respect to AFz (REF) and referred to Fpz (GND). Analog signals are conditioned by stages of amplification and filtering (*Analog Filter and Amplifier*). Then, they are digitized by the Analog Digital Converter *ADC* and sent by the *Wireless Transmission Unit* to the *Data Processing* block. A 12-bands *Filter Bank* and a *Common Spatial Pattern* (*CSP*) algorithm carry out the feature extraction. The *Classifier* receives the feature arrays and detects the emotional valence.

### Data processing

In this section, the *features extraction and selection* and the *classification* procedures of the proposed method are presented.

#### Features extraction and selection

Finer-resolution partitions of the traditional EEG bands were proposed for emotion recognition^67,68^. In the present work, a 12-band Filter Bank version, recently adopted in distraction detection^69^, is employed.

Spatial and frequency filtering is applied to the output data of the filter bank. A well-claimed Common Spatial Pattern (CSP), widely used in EEG-based motor imagery classification^70–73^, is used as a spatial filter. For the first time, the FB-CSP pipeline is here proposed in the field of valence emotion detection.

A previous study^74^ showed that the CSP spatial filtering method entails the relationship between EEG bands, EEG channels, neural efficiency and emotional stimuli types. It demonstrated that CSP spatial filtering gives significant values on band-channels (p < 0.004) combination. Spatial characteristics may provide more relevant information to distinguish different emotional states. A feasibility study demonstrated the CSP capability of applying spatial features to EEG-based emotion recognition reaching average accuracies of 85.85 % and 94.13 % on the self-collected and MAHNOB-HCI datasets. Three emotion tasks were detected with 32 EEG channels^75^.

In a binary problem, the CSP computes the covariance matrices of the two classes. By means of a whitening matrix, the input data are transformed in order to have an identity covariance matrix (mainly, all dimensions are statistically independent). Resultant components are sorted on the basis of variance in order: (i) *decreasing*, if the projection matrix is applied to inputs belonging to class 1, and (ii) *ascending*, in case of inputs belonging to class 2. In this way, according to the "variance of each component", data can be more easily separable^76^. The CSP receives as input 3D tensors with dimensions given by the number of channels, filters, and samples.

#### Classification

In this study, the emotional valence is classified using a *k*-Nearest Neighbors (*k*-NN)^77^ for cross-subject case and full-connected Artificial Neural Networks (ANNs)^78^ for within-subject one. One of the main advantages of the *k*-NN is that, being non-parametric, it does not require a training phase unlike other Machine Learning methods. In a nutshell, given a set of unlabelled points *P* to classify, a positive integer *k*, a distance measure *d* (e.g., Euclidean) and a set *D* of already labelled points, for each point $p \in P$, *k*-NN assigns to *p* the most frequent class among its *k* neighbours in *D* according to the measure *d*. The number of neighbours *k* and the distance measure *d* were set using a cross-validation procedure. Differently from *k*-NN, ANNs are classification models that require a training procedure. In general, an ANN consists of a set of elements (called *neurons*) arranged together into several layers fully connected between them. Each neuron performs a linear combination of its inputs usually followed by the application of a non-linear function called *activation function*. It was demonstrated^79^ that an ANN can approximate arbitrarily complex functions, giving to the model the ability to discriminate between different classes. The number of neurons, the number of layers and the activation functions are hyperparameters given a priori, while the coefficients of each linear combination are learned by the model in a training stage.

## Experiments and results

### Data acquisition setup

The experimental protocol was approved by the ethical committee of the University Federico II. Written informed consent was obtained by the subjects before the experiment. All methods were carried out in accordance with relevant guidelines and regulations. Prior informed consent for publication of identifying information and images was obtained by all the participants. Thirty-one volunteers, not suffering from both physical and mental pathologies, were screened by means of the Patient Health Questionnaire (PHQ) for excluding depressive disorders^80^. Six participants were excluded from the experiment owing to their score in PHQ, resulting in twenty five healthy subjects, (52 % male, 48 % female, aged 38 ± 14). The experiments were conducted in a dark and soundproofed environment to prevent disturbing elements.

**Figure 2:**
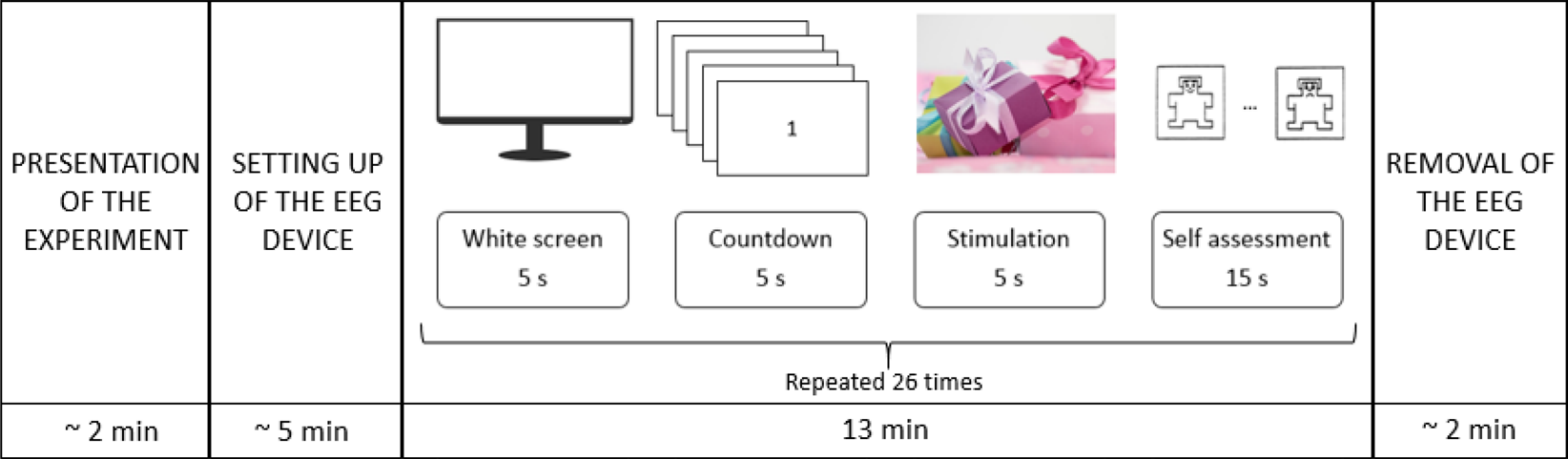
Experimental protocol.

The employed Mood Induction Procedure (MIP) was based on the presentation of emotion-inducing material to participants to elicit suitable emotions. The subjects were instructed on the purpose of the experiment. They had to passively gaze at the pictures projected on the screen and, only after, to assess the experienced valence by two classes: negative and positive. Emotional stimuli were presented without explicitly instructing subjects to get into the suggested mood state and regulate their emotions. Nevertheless, the subjects were aware of both the elicitation stimulus and the type of induced emotion (although it was not explicitly stated, they could guess it starting from the self-assessment questionnaire). Thus, the employed task was of a type *implicit-more controlled*^81^. The experiment was made of 26 trials. Each trial lasted 30 s and consisted of: (i) a 5-s white screen, (ii) a 5-s countdown frame employed to relax the subject and separate emotional states mutually, (iii) a 5-s elicitative image projection, and (iv) a 15-s self-assessment (Fig. 2). The subject was required to express a judgement on the positivity/negativity of his/her valence on a scale from 1 to 5 through the self-assessment manikin (SAM) questionnaire. In each trial, different images were projected, for a total of 26 images. 13 pictures for eliciting negative valence and 13 for eliciting positive valence were employed. Positive and negative tasks were randomly administered to participants in order not to create expectations in the tested subjects.

Images were chosen from the reference database Oasis^82^. Oasis attributes a valence level to each image on a scale from 1.00 to 7.00.

**Figure 3:**
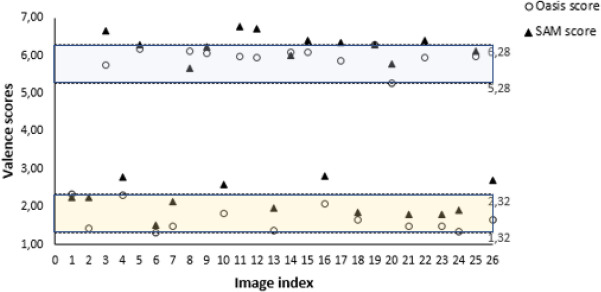
Oasis valence score and SAM average scores of the 26 images selected for the experiments. The Oasis score intervals used to extract polarized images are identified by dotted lines.

Only Italian volunteers participated the experiment, thus a pre-test on the trans-cultural robustness of the selected images was administered to a different group consisting of 12 subjects. Specifically, suitable pictures were shown and was asked subjects to rate each image using the scale "self assessment manikin" (SAM). Images with a neutral rating from at least 50 % of the subjects were excluded from the experiment. In fact, a stimulus strongly connoted in a specific cultural framework, loses its strength out of that context. An emblematic example are the symbols related to the Ku Klux Klan. Those have a different connotative richness for a citizen of the United States of America compared to European people. The same pre-test revealed very low performances for detecting valence level when the stimuli score was around the the midpoint value of the valence scale. The sensitivity of the system was improved by selecting a suitably polarised subset of Oasis images, as in^53^ and^57^. First of all, images with highest and lowest valence score were identified: respectively 6.28 and 1.32. Then, 1.00 was the span chosen to guarantee the trade-off between the maximum image polarization and an adequate quantity of images to build the experiment (>100). Therefore, [1.32, 2.32] and [5.28, 6.28] were adopted as the scoring intervals for negative and positive stimuli valence, respectively and 13 images per group were randomly selected. For each image, the Oasis valence score and the average scores (on all subjects) of the self-assessment are shown in Fig. 3. The maximum difference between the SAM and the stimuli scores is lower than the average standard deviation (1.00) computed on the Oasis scores.

The number of images per class was chosen in order to guarantee a trade-off between the amount of experimental epochs and the user comfort, by minimizing the duration of the experiment simultaneously. In this way, the experiment lasted about 20 min per subject. About 2 min were required for the presentation of the activity to the subject, other 5 min were required for the setting up of the EEG device quality. 13 min were required for the completion of all the 26 trials.

**Figure 4:**
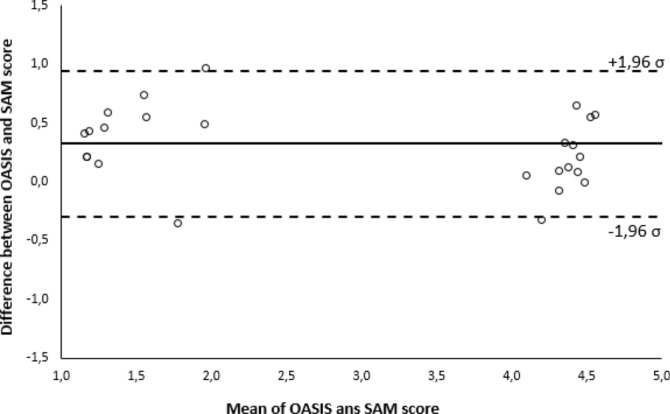
Bland-Altman analysis on the agreement between stimuli (OASIS) and volunteers perception (SAM).

Bland-Altman and Spearman analyzes were carried out to compare the experimental sample with respect to the Oasis experimental sample. The agreement between the measurements expressed by the two samples is verified, as evidenced by a qualitative analysis in Fig. 4 and the Spearman correlation index $\rho =0.799$.

### Hardware

**Figure 5:**
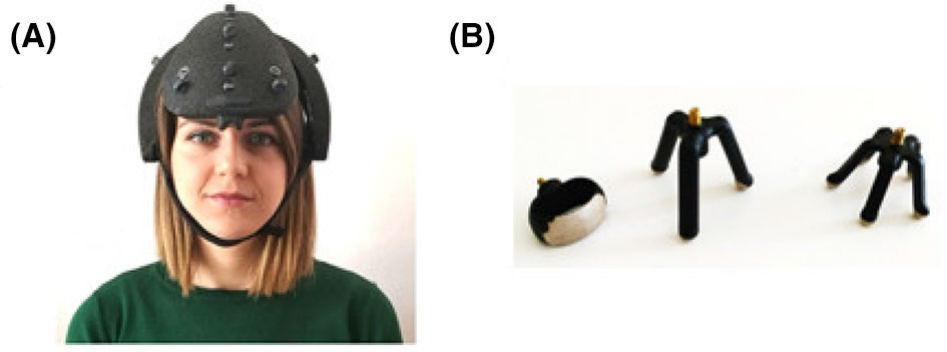
(**A**) EEG data acquisition system *Helmate8* and (**B**) dry electrodes from *abmedica*.

The position of the used channels was chosen by taking into account the well-assessed theories of emotions already presented: frontal asymmetry and right hemisphere asymmetry^20–22,25^. The *ab medica Helmate*^83^ was found to fit the requirements of the previous mentioned theories because it is equipped with 3 frontal, central, and occipital channels pairs. Indeed, the coverage of almost all areas of the scalp ensured that both frontal and hemispheric asymmetries were recorded, despite the low number of electrodes. The device provided electrodes placed on Fp1, Fp2, Fz, Cz, C3, C4, O1, and O2, according to the 10/20 International Positioning System. The Helmate is Class IIA certified according to Medical Device Regulation (UE) 2017/745 (Fig. 5A). It is provided with a rechargeable battery and is able to transmit the acquired data via Bluetooth, without connection cables. This ultra-light foam helmet is equipped with 10 dry electrodes which 8 acquisition channels (unipolar configuration) and with disposable accessories (under-helmet and under-throat). Electrodes are made of conductive rubber and their endings are coated with Ag/AgCl. They have different shapes to pass through the hair and reach the skin (Fig. 5B).

The resulting signals are recorded differentially vs ground (Fpz), and then referenced with respect to AFz, both placed in the frontal region. A dedicated software measures the contact impedance between the electrodes and the scalp. The acquired EEG signal, sampled at 512 Hz, is sent to the Helm8 Software Manager. It allows both to display the signal directly on PC in real time and to apply a large variety of pre-processing filters. The device has an internal $\mu$ SD for backup purposes. Helmate incorporates a Texas Instruments analog front-end, the ADS1298^84^. This is a multichannel, simultaneous sampling, 24-bit, ($\Delta \Sigma$) analog-to-digital converter (ADCs) with built-in programmable gain amplifiers (PGAs), internal reference, and an onboard oscillator. Main features of the ADS1298 are: (i) eight Low-Noise PGAs and Eight High-Resolution ADCs; (ii) input-Referred Noise: 4 $\mu$VPP (150 Hz BW, G = 6); (iii) input Bias Current: 200 pA; and, (iv) CMRR: –115 dB.

### Data processing comparison

The EEG tracks were acquired at a sampling frequency of 512 Hz and filtered between 0.5 and 48.5 Hz using a zero-phase 4$^{th}$-order digital Butterworth filter. In the processing stage, the used trials resulted to be 24 for each subject since macroscopic artifacts corrupted one trial of three subjects. So, to keep the dataset balanced, the number of trials was reduced by removing the compromised trial and another one randomly chosen among those of the opposite class. Then, for the remaining subjects, two trials of different classes were randomly removed to guarantee the same amount of data for all the participants. The remaining artifacts were removed from EEG signals using Independent Component Analysis (ICA) by means of the EEGLAB Matlab toolbox version 2019^85^. The recorded EEG signals were divided into 2 s time windows overlapping of 1 s.

The traditional EEG bands delta (0-4 Hz), theta (4-8 Hz), alpha (8-13 Hz), beta (13-30 Hz), and gamma (>30 Hz) were extracted. The proposed method was validated by comparing different approaches of features extraction and classification. For EEG features extraction, two different methods were adopted: (i) with and (ii) without a priori spatial-frequency knowledge provided by neurophysiology.

In a-priori spatial knowledge framework, frontal asymmetry feature was chosen, computed by subtracting the left frontal (FP1) from the right (FP2) channel. Moreover, the whole hemispherical asymmetry was also considered and the differences of the three symmetric channel pairs were input to the classifiers. The analysis considered only spatial or both spatial and frequency features, according to the different neurophysiological theories. A-priori frequential knowledge led to the use of a [8-13] Hz (alpha band) pass-band filter (zero-phase 4$^{th}$-order digital Butterworth filter).

Without a priori knowledge, features were extracted via the PCA and CSP algorithms. For PCA, we used a number of components which explains the 95 % of the data variance. For CSP, all the 96 components returned by the algorithm are used. Also in this case, only the spatial information and the combination of spatial and frequency information were analysed. Input features were 8192 (8 channels * 1024 samples) when PCA and CSP were fed only by spatial information.

The acquired EEG signal was filtered through 12 IIR band-pass filters Chebyshev type 2, with 4 Hz bandwidth, equally spaced from 0.5 to 48.5 Hz. In this way, the traditional five EEG bands (delta, theta, alpha, beta, and gamma) are divided into 12 sub-bands. Therefore, the features resolution is increased by the narrowing of the bands. Thus, features increased to 98304 (12 frequency bands * 8 channels * 1024 samples). The features were then reduced from 98304 to 96 using the CSP algorithm.

Subsequently, in the classification stage, two types of investigations were carried out: within-subject and cross-subject. In the first case, data of a single subject were employed for training and classification phases, while in the second one, the data set as a whole was employed. In both cases, the proposed method was validated through a stratified 12-fold Cross Validation (CV) procedure. Namely, given a combination of the classifier hyperparameters values, a partition of the data composed of *K* subsets (folds) is made, preserving the ratio between the samples of different classes. A set *T* consisting of $K-1$ folds is then used to train the model and, when required, the CSP projection matrix; the remaining fold *E* to measure the model performances using any metric scores (e.g., accuracy). The whole process is then repeated for all the possible combinations of the *K* folds. Finally, the average scores on all the test sets are reported. Furthermore, training and test sets are made keeping together the epochs of each trial (consisting of 4 epochs each) in the same set, both in the cross-subject and in the within-subject approach. In this way, the training and the test sets do not include parts of the same trial. Finally, in a 12-fold scheme within-subject setup, 88 epochs for training and 8 epochs for testing are used. Of the 88 epochs used for the training set, 16 are exploited as validation set in the ANNs learning. Instead, in the cross-subject case, considering that the experimental campaign involved 25 subjects, a total of 2400 epochs was used. This, in a 12-fold cross validation scheme, corresponds to 2200 epochs as training test and 200 epochs as test set. In the ANNs learning, 200 epochs are used as the validation set.

**Figure 6:**
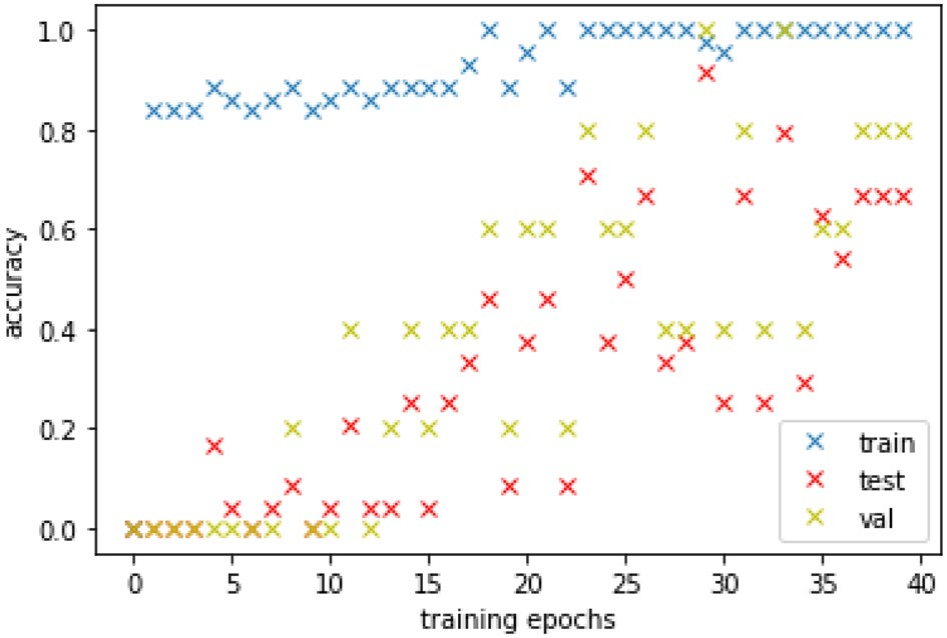
First 40 ANN training epochs on one subject.

*k*-NN^77^ and ANN^78^ were compared with other four classifiers: Linear Discriminant Analysis (LDA)^86^, Support Vector Machine (SVM)^87^, Logistic Regression (LR)^78^ and Random Forest (RF)^73^. LDA searches for a linear projection of the data in a lower dimensional space trying to preserve the discriminatory information between the classes contained in the data. A SVM defines a separator hyperplane between classes exploiting a subset of the training instances (support vectors). LR is a widely used classification method based on the logistic function. In binary classification, it estimates the probability of a sample $\mathbf {x}$ to belong to a class labelled as $y=1$ as $P(y|\mathbf {x})=\frac{\exp (q +\mathbf {wx})}{1+\exp (q +\mathbf {wx})}$ where $\mathbf {w}$ and *q* are learnable parameters. A RF combines several decision trees to make classifications. The use of several decision tree helps in improving the accuracy. Furthermore, to prevent possible over-fitting, regularization terms in the training procedures were used for SVM learning using the SVM soft-margin formulation^87^, and for neural networks learning using a weight decay^88^ during the learning algorithm execution. ANNs were trained with the ADAM algorithm. A maximum number of 1000 epochs with a patience of 50 epochs on the validation set was used to train the network models. Figure 6 shows the trend of the accuracy during the first 40 iterations of a learning stage on a single subject model. For all the classifiers, the hyperparameters used during the CV procedure are reported in Table 2. Accuracy, precision, and recall are reported to assess the classification output quality. Precision measures result relevancy, while recall how many truly relevant results are returned. The F1 score, combining precision and recall, was computed to assess the classification performance in minimizing false negatives for the first class (negative valence) analysis. Considering many use cases, the minimization of failure in recognizing negative valence is the main issue.

**Table 2:** Classifier optimized hyperparameters and variation range.

Classifier	Hyperparameter	Variation Range
*k*-nearest neighbour (*k*-NN)	Distance (DD)	{cityblock, chebychev, correlation, cosine, euclidean, hamming, jaccard, mahalanobis, minkowski,spearman}
	DistanceWeight (DW)	{equal, inverse, squaredinverse}
	Exponent (E)	[0.5, 3]
	NumNeighbors (NN)	[1, 5]
Support vector machine (SVM)	BoxConstraint (BC)	log-scaled in the range [1e-3,1e3]
	KernelFunction (KF)	{gaussian, linear, polynomial}
	KernelScale (KS)	log-scaled in the range [1e-3,1e3]
	PolynomialOrder (PO)	{2,3,4}
Artificial neural network (ANN)	Activation Function (AF)	{relu, sigmoid, tanh}
	Hidden layer nr. of neurons (HLN)	[25, 200]
Linear discriminant analysis (LDA)	Gamma (G)	[0,1]
	Delta (D)	log-scaled in the range [1e-6,1e3]
	DiscrimType (DT)	{linear, quadratic, diagLinear,}
		{diagQuadratic, pseudoLinear, pseudoQuadratic}
Random forest (RF)	Depth (D)	[5,20]
	Number of trees (NT)	[15,100]
	Maximum depth of the tree	[5,30]
Logistic regression (LR)	Penalty (P)	{L2, elastic net}
	Inverse of regularization strength (C)	[0.25, 1.0]

### Experimental results

Accuracy was related to the model’s ability to correctly differentiate between two valence states. EEG tracks relating to the negative and positive image tasks were associated to the first and the second class, respectively.

**Figure 7:**
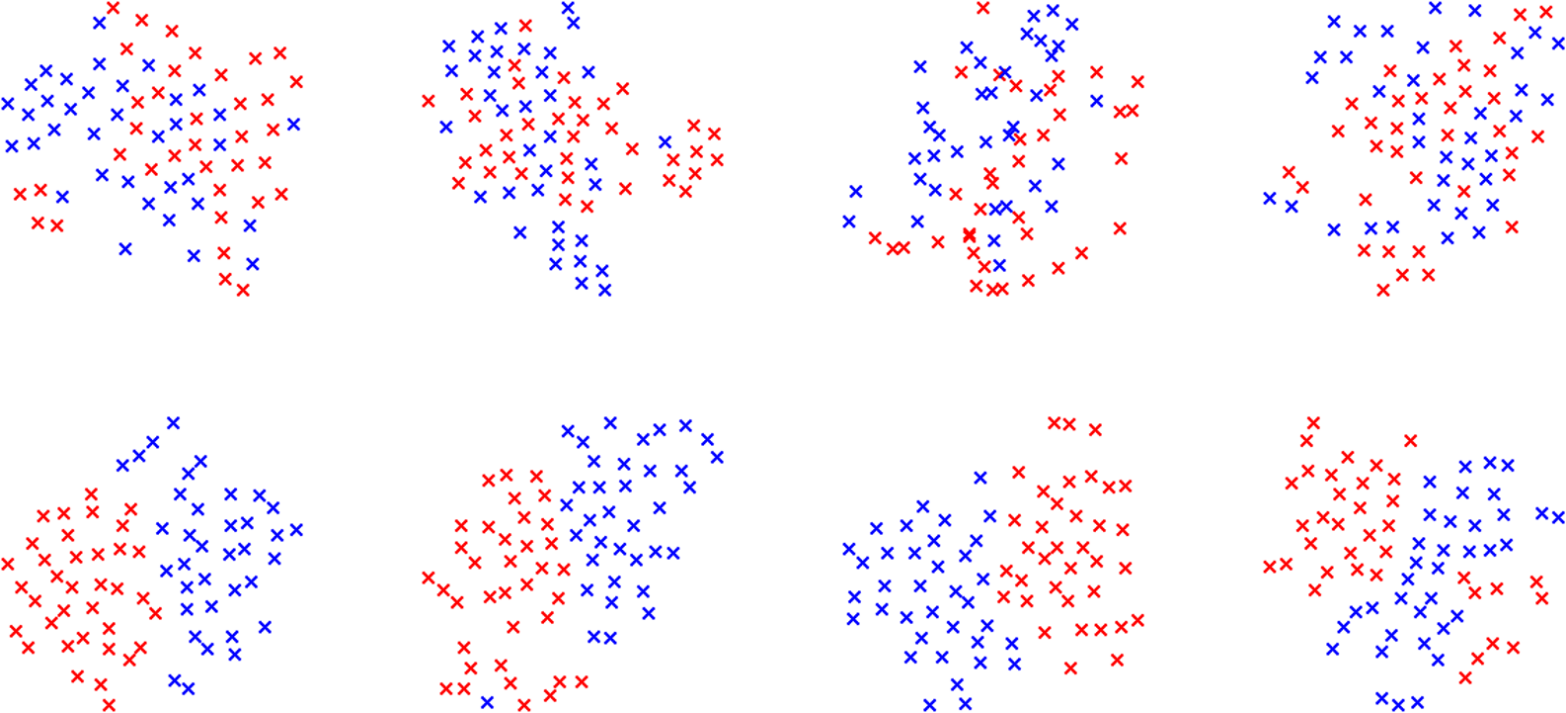
t-SNE based data comparison of four random subjects projected in the CSP space, without (first row) and with (second row) the Filter Bank. Filter Bank improves the classes (blue and red) separability.

The mean of the individual accuracies and standard deviations computed on each subject (within-subject case) and the accuracies and standard deviations computed on all subjects data as a whole (cross-subject case) are showed when a priori spatial-frequency knowledge is used (Table 3) or not (Tables 4 and 5). Results are shown at varying the adopted classifier. Better performances are obtained without a-priori knowledge and when features are extracted by combining Filter-Bank and CSP, both in within-subject and cross-subject case. In within-subject analysis, the data subsets are more uniform and all the classifiers provide very high accuracy. In Fig. 7 the data of four random subjects projected in the CSP space, with and without the Filter Bank, are compared. The classes, after using the Filter Bank, are easily separable with respect to the use of the only CSP, as highlighted by the results. In Table 6, the accuracies in the within-subjects experiments are reported for all the subjects. In cross-subject analysis, when data from all subjects are merged, variability increases and not all the classifiers give good results. Interestingly, in the cross-subject approach, the *k*-NN classifier allows to achieve by far the best performance, while the scores degrade using the other classifications setups. This behaviour suggests that the data of similar classes are close together for different subjects, but that in general they are not easily separable through classical Machine Learning methods. Moreover, a feature selection analysis using the Mutual Information (MI) method, proposed in^89^, was made using the best experimental setups of both within-subject and cross-subject approaches. The results reported in Table 7 show that just the 12.5 % of the FBCSP features are enough to achieve accuracy performances over the 90 % in the within-subject case. Therefore, the features extracted by the CSP in conjunction with Filter Bank resulted effective in emotional valence recognition.

**Figure 8:**
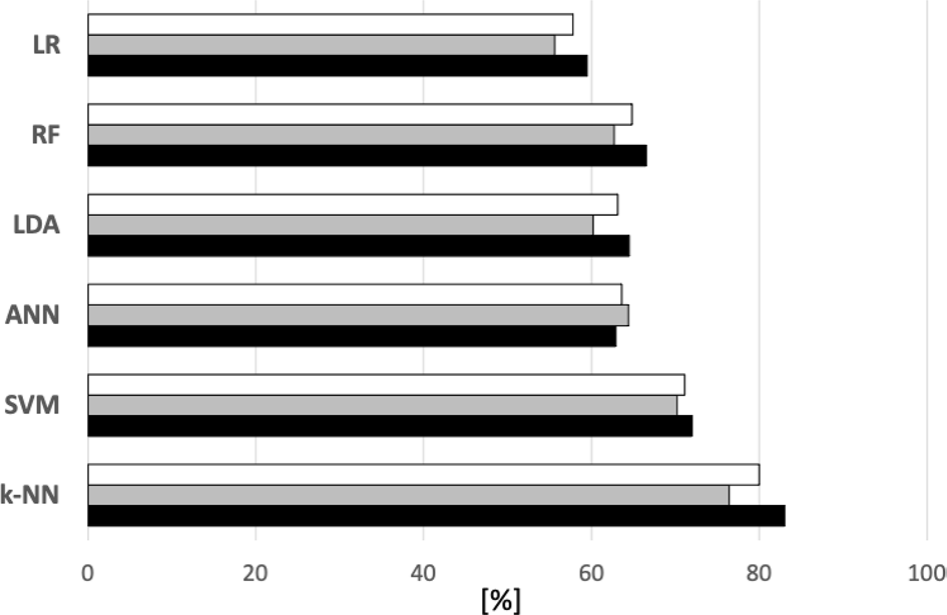
F1-score (White), Recall (Grey) and Precision (Black) for the best performance of each classifier—cross-subject.

In conclusion, the proposed solution based on 12-bands Filter-Bank provides the best performances reaching 96.1 % of accuracy with ANN in within-subject analysis and 80.2 % using *k*-NN with $k=2$ in cross-subject analysis. In the within-subject case, for the ANN the best top-5 subjects reached the best performances using ANN with one layer with less than 100 neurons equipped with the classical *tanh* activation function, showing that networks with few parameters can be sufficient to address this classification problem as long as a proper set of features is provided. Precision, Recall and F1-score metrics are reported in Fig. 8.

**Table 3:** Accuracy (mean and standard deviation) considering a priori knowledge i.e. asymmetry—within-subject (Within) and cross-subject (cross).

Classifier	Entire EEG Band		$\alpha$band	
	Within	Cross	Within	Cross
*k*-NN	54.0 ± 4.1	51.0 ± 1.2	53.8 ± 4.0	51.3 ± 0.4
SVM	56.8 ± 3.4	50.8 ± 0.2	56.7 ± 3.0	51.2 ± 0.3
LDA	54.5 ± 3.8	51.2 ± 0.8	53.8 ± 3.5	51.0 ± 1.0
ANN	58.3 ± 3.0	51.8 ± 0.3	58.5 ± 3.0	51.5 ± 1.6
RF	55.7 ± 3.9	50.7 ± 1.2	54.5 ± 4.5	50.9 ± 1.3
LR	52.5 ± 4.1	51.4 ± 0.2	53.7 ± 4.3	51.2 ± 0.7

**Table 4:** Accuracy (mean and standard deviation) without considering a priori knowledge i.e. Asymmetry - Within-subject. The best performance value is highlighted in bold.

Classifier	Entire EEG band			Filter bank		
	No PCA/CSP	PCA	**CSP**	No PCA/CSP	PCA	CSP
*k*-NN	71.0 ± 6.0	67.7 ± 8.4	72.0 ± 8.9	75.6 ± 5.8	66.8 ± 7.2	94.5 ± 3.5
SVM	66.9 ± 8.1	66.3 ± 10.3	73.4 ± 9.5	71.6 ± 8.9	62.0 ± 7.8	95.5 ± 2.8
LDA	63.1 ± 4.9	55.3 ± 4.0	74.0 ± 10.0	62.9 ± 5.3	53.9 ± 3.5	95.0 ± 2.9
ANN	69.7 ± 5.1	66.3 ± 6.2	78.1 ± 8.0	66.7 ± 4.9	65.6 ± 5.6	**96.1 ± 3.0**
RF	$66.4\pm 4.1$	$58.9\pm 4.2$	$72.8\pm 9.4$	$67.4\pm 4.1$	$59.3\pm 5.0$	$94.2\pm 2.7$
LR	$62.7\pm 4.9$	$52.3\pm 2.9$	$72.6\pm 9.3$	$61.0\pm 5.0$	$51.2\pm 4.0$	$95.1 \pm 2.9$

**Table 5:** Accuracy (mean and standard deviation) without considering a priori knowledge i.e. Asymmetry - Cross-subject. The best performance value is highlighted in bold.

Classifier	Entire EEG Band			Filter Bank		
	No PCA/CSP	PCA	SP	No PCA/CSP	PCA	CSP
*k*-NN	68.4 ± 0.2	62.1 ± 0.9	56.8 ± 0.5	70.1 ± 1.0	61.1 ± 0.3	**80.2 ± 2.1**
SVM	51.5 ± 0.6	52.1 ± 0.3	61.0 ± 2.0	51.8 ± 1.0	51.2 ± 0.3	71.3 ± 2.0
LDA	53.5 ± 0.7	50.9 ± 0.4	55.4 ± 4.2	52.6 ± 0.1	50.9 ± 0.2	63.7 ± 2.1
ANN	59.9 ± 1.0	54.5 ± 0.2	58.1 ± 1.1	57.4 ± 0.1	53.7 ± 0.1	63.3 ± 2.7
RF	$56.5\pm 0.6$	$55.3\pm 0.7$	$59.2\pm 1.9$	$57.8\pm 1.1$	$52.5\pm 2.9$	$65.0 \pm 3.8$
LR	$50.5\pm 1.9$	$50.6\pm 0.5$	$55.7\pm 4.9$	$51.8\pm 0.9$	$50.9 \pm 0.5$	$58.1 \pm 1.5$

**Table 6:** Accuracies obtained for each subject in the within-subject experiments when a FB-CSP pipeline is adopted.

Subject	*k*-NN	SVM	LDA	ANN	RF	LR
#1	95.8	95.8	94.4	95.8	93.3	94.4
#2	95.8	92.2	92.2	93.1	92.2	95.8
#3	94.4	93.6	93.7	94.4	91.1	92.2
#4	95.8	98.6	98.1	99.0	94.4	94.4
#5	91.7	93.8	93.2	94.4	93.1	93.1
#6	97.2	96.2	95.8	97.2	93.9	95.8
#7	95.8	96.1	95.8	98.6	94.4	95.8
#8	97.2	98.6	97.2	99.0	97.2	97.0
#9	98.6	98.6	98.6	98.6	96.2	98.6
#10	92.0	94.6	94.4	97.2	95.8	94.5
#11	95.8	95.0	94.6	97.2	93.6	95.0
#12	94.4	94.7	94.4	97.2	92.3	94.4
#13	98.6	98.6	98.6	99.0	95.8	98.6
#14	95.8	95.7	95.8	95.8	97.2	94.4
#15	85.9	91.2	90.5	91.0	89.9	90.3
#16	95.4	97.2	96.7	98.2	97.2	97.0
#17	86.3	95.0	94.6	93.1	92.7	95.8
#18	93.1	91.4	90.2	92.0	92.7	93.0
#19	97.2	98.6	98.7	99.0	98.6	98.6
#20	94.4	98.5	97.2	95.8	95.4	97.2
#21	97.2	97.2	97.2	98.6	95.8	98.6
#22	98.6	97.9	97.4	99.0	95.4	97.2
#23	89.3	90.0	89.2	89.4	88.1	88.9
#24	95.2	97.9	97.3	98.7	98.6	98.3
#25	90.2	90.1	89.4	90.3	90.2	88.5
*Average * ± * std.*	94.4 ± 3.5	95.5 ± 2.8	95.0 ± 2.9	96.1 ± 3.0	94.2 ± 2.7	95.1 ± 2.9

**Table 7:** Accuracy performances of the best processing solutions for both within- and cross-subject approaches at varying the number of input features selected through the mutual information strategy.

Classifier		#Features			
		12	24	50	96
*k*-NN	Cross	$58.7 \pm 1.0$	$65.1 \pm 1.8$	$74.4 \pm 0.9$	$80.2 \pm 2.1$
ANN	Within	$92.8 \pm 4.1$	$93.0 \pm 4.1$	$93.4 \pm 1.0$	$96.1 \pm 3.0$

### Discussion

In the previous Sections the measurability foundation of emotion was discussed. In this study, results from the *Self Assessment Manikin* questionnaire confirmed the compatibility of the experimental sample with that of *Oasis* thus improving the reproducibility of the experiment and the generalizability of the outcome. Moreover, the reference theory adopted allows the measurement of emotions arranging them along interval scales. In this framework, the preliminary binary classification of the proposed system could be enhanced by increasing the number of classes. Thus, the number of valence states increase and a higher resolution metric scale can be obtained. Therefore, the Circumplex Model is compatible with an upgrade of the proposed binary classification method. It is noteworthy that the number of classes can increase if emotional valence states can be experimentally induced at higher resolution. This is precisely what the standardized stimuli datasets allow because their scores are organised according to an interval scale. The novelty of this research is based on the compliance with different quality parameters. In Table 8, this study is compared with the works examined in Section 2 section, taking into account the following criteria: (i) classification vs measurement, (ii) standardized stimuli, (iii) self-assessment questionnaires, (iv) number of channels $\le 10$, (v) cross-subject accuracy > 80 % (vi) within-subject accuracy > 90 %. As concerns the first quality parameter, the option between classification and measurement is related to the reference theory adopted (i.e., discrete model or circumplex model).

There are only two studies combining SAM and standardized stimuli ratings for the construction of the metrological reference^61,63^. Therefore, literature concerning EEG-based emotion detection exhibits a lack of generalizability for the presented results. Among all the examined works, the proposed study is the only one that matches all the aforementioned criteria.

**Table 8:** Studies on emotion recognition classified according to metrological approach, number of channels and accuracy (n.a. = "not available", $\checkmark$ = "the property is verified". Only for the first line, $\checkmark$ = "Measurement" ).

	^34^	^35^	^28^	^36^	^37^	^29^	^30^	^38^	^39^	^40^	^55^	^41^	^42^	^31^	^43^	^52^	^44^	^32^	^33^	^56^	^57^	^58^	^59^	^60^	^53^	^61^	^62^	^63^	^64^	**Our work**
Measurement vs Classification	$\checkmark$	$\checkmark$	$\checkmark$	$\checkmark$	✗	$\checkmark$	✗	$\checkmark$	$\checkmark$	$\checkmark$	✗	$\checkmark$	$\checkmark$	$\checkmark$	✗	✗	$\checkmark$	$\checkmark$	$\checkmark$	$\checkmark$	$\checkmark$	✗	✗	$\checkmark$	$\checkmark$	$\checkmark$	✗	$\checkmark$	$\checkmark$	$\checkmark$
Standardized Stimuli	✗	✗	✗	✗	✗	✗	✗	✗	✗	✗	✗	$\checkmark$	$\checkmark$	✗	✗	✗	✗	✗	$\checkmark$	✗	$\checkmark$	✗	✗	✗	$\checkmark$	$\checkmark$	✗	$\checkmark$	$\checkmark$	$\checkmark$
Self-assessment Questionaries	$\checkmark$	$\checkmark$	$\checkmark$	$\checkmark$	$\checkmark$	$\checkmark$	$\checkmark$	$\checkmark$	$\checkmark$	$\checkmark$	$\checkmark$	$\checkmark$	$\checkmark$	$\checkmark$	$\checkmark$	$\checkmark$	$\checkmark$	$\checkmark$	$\checkmark$	$\checkmark$	$\checkmark$	$\checkmark$	$\checkmark$	$\checkmark$	✗	$\checkmark$	n.a.	$\checkmark$	$\checkmark$	$\checkmark$
#channels$\le 10$	✗	✗	✗	✗	✗	✗	✗	✗	✗	✗	$\checkmark$	✗	✗	✗	✗	✗	✗	✗	✗	$\checkmark$	$\checkmark$	$\checkmark$	$\checkmark$	$\checkmark$	✗	$\checkmark$	$\checkmark$	$\checkmark$	$\checkmark$	$\checkmark$
Cross-subject Accuracy (>80%)	✗	$\checkmark$	$\checkmark$	$\checkmark$	$\checkmark$	$\checkmark$	$\checkmark$	n.a	$\checkmark$	$\checkmark$	n.a.	n.a	n.a	n.a	n.a	n.a	n.a	$\checkmark$	$\checkmark$	n.a	$\checkmark$	n.a	$\checkmark$	✗	✗	$\checkmark$	✗	✗	✗	$\checkmark$
Within-subject Accuracy (>90%)	n.a.	n.a.	$\checkmark$	n.a.	n.a.	n.a.	n.a.	✗	n.a.	n.a.	$\checkmark$	$\checkmark$	$\checkmark$	$\checkmark$	✗	$\checkmark$	✗	n.a.	$\checkmark$	✗	n.a.	$\checkmark$	n.a.	n.a.	n.a.	n.a.	n.a.	n.a.	$\checkmark$	$\checkmark$

## Conclusion

An emotional-*valence* detection method for an EEG-based system was proposed by proving experimentally accuracy of 96.1 % and 80.2 % in within-subject and cross-subject analysis, respectively. Important steps towards the measurability of emotions were proposed: Firstly, the Valence detection occurs along the interval scale theorized by the Circumplex Model of emotions. Thus, the current binary choice, positive valence vs negative valence, could represent a first step towards the adoption of a metric scale with a finer resolution. Secondly, the experimental sample was collected by managing the bias of depressive disorders. Finally, results from the *Self Assessment Manikin* questionnaire confirmed the compatibility of the experimental sample with that of *Oasis*. Hence, a metrological reference was built taking into account both the statistical strength of the data set OASIS and the collected data about the subject perception. The OASIS dataset was also subjected to a cross-cultural validity check. A priori information is not needed using algorithms capable of extracting features from data through an appropriate spatial and frequency filtering. Classification is carried out with a time window of 2 s. The achieved performances are due to the combined use of a custom 12-band Filter Bank with CSP spatial filtering algorithm. This approach is widely used in the motor imagery field and was proven to be valid also for emotion recognition. In the future, it would be interesting to test the FB-CSP approach also on public datasets. The high ergonomics and accuracy are compatible with the principal applications of emotional valence recognition. Future developments of the research will be: (i) the development of the metrological foundation of emotion measurement (theoretical model, measurement unity, uncertainty analysis); (ii) a resolution improvement of the valence metric scale; (iii) addition of arousal assessment to the detection of emotional valence, (iv) combined use of different biosignals (besides EEG); (v) a deep analysis on interactions among the number of electrodes, classifiers, and the accuracy; and (vi) experiments on different processing strategies: in this study, the binary nature of the problem enhanced the classification performances of the k-NN. In future works aimed at increasing the metric scale resolution, other methods may result more effective (SVM, full-connected neural networks, Convolutional Neural Networks^62^ etc.) for example in a regression-based perspective.
